# The expected and actual communication of health care workers during the management of intrapartum: An interpretive multiple case study

**DOI:** 10.4102/phcfm.v7i1.911

**Published:** 2015-12-03

**Authors:** Doreen K.M. M'Rithaa, Sue Fawcus, Mikko Korpela, Retha De la Harpe

**Affiliations:** 1 Information Technology, Cape Peninsula University of Technology, South Africa; 2 Department of Interdisciplinary Health Sciences, Stellenbosch University, South Africa; 3 Mowbray Maternity Hospital, South Africa; 4 Obstetrics and Gynaecology, University of Cape Town, South Africa; 5 School of Computing, University of Eastern Finland, Finland

## Abstract

**Background:**

Daily activities within a health care organisation are mediated by information communication processes (ICP) involving multiple health care professionals at different levels of care. Effective perinatal management requires critical information to be accurately communicated. If there is a breakdown in this communication patient safety is at risk for various reasons such as: inadequate critical information, misconception of information and uninformed decisions being made. The purpose of this study was to interpret the complexities around ICP in order to contribute to the effective management of the intrapartum period.

**Methods:**

Multi method, multiple case study approach was used to understand the ICP during the management of the intrapartum period. During the study, the expected ICP, the actual ICP, the challenges involved and the desired ICP were analysed. Twenty-four in-depth interviews with skilled birth attendants (SBAs) employing observer-as-participant roles, field notes, and document review methods were utilised to gather the data. Thematic analysis was utilised to analyse the data using Atlas TI software.

**Results:**

The study revealed three subthemes which emerged from the expected ICP, whilst three others that emerged formed the theme actual ICP. The subthemes from the expected ICP included: accessibility of obstetric services, expected referral, recommended tools, expected communication and expected documentation. The theme actual ICP held three emerging subthemes: the handover processes, collaborative information seeking, information communicated and referral processes.

**Conclusion:**

This study showed that what was expected was not what was actually happening. The requirements of the policies and protocols need to be effectively implemented to improve practice building these into current biomedical guidelines.

## Abstrait

**La communication attendue et effective entre les travailleurs de santé pendant la gestion des soins périnatals: une étude interprétative de cas multiples**.

**Contexte:**

Les activités journalières au sein d'un établissement de santé sont influencées par le processus de communication des informations (PCI) impliquant de nombreux professionnels de la santé aux différents niveaux de soins. La prise en charge périnatale efficace exige une communication précise des informations essentielles. S'il y a un manque de communication la sécurité des patients est en danger pour plusieurs raisons: des informations essentielles inadéquates, des malentendus sur les informations et des décisions malavisées peuvent être prises. Le but de cette étude était de relever les complexités du PCI afin de contribuer à la prise en charge efficace de la période périnatale.

**Méthodes:**

On a utilisé une étude multi-méthode de plusieurs cas pour comprendre le PCI pendant la prise en charge de la période périnatale. Au cours de l’étude, le PCI attendu, le PCI effectif, l'enjeu et le PCI désiré ont été analysés. Pour collecter les données on a eu vingt-quatre entrevues en profondeur avec des sages-femmes qualifiées (SFQ) au moyen de rôles d'observateur participant, des notes de terrain, et des méthodes de revue des documents. On s'est servi de l'analyse thématique pour analyser les données au moyen du logiciel Atlas Ti.

**Résultats:**

L’étude a mis en évidence trois sous-thèmes qui sont ressortis du PCI attendu, alors que les trois autres qui sont apparus forment le thème du PCI effectif. Les sous-thèmes du PCI attendu sont: l'accessibilité des services obstétriques, l'orientation attendue, les outils recommandés, la communication attendue et la documentation attendue. Le thème effectif du PCI contenait trois sous-thèmes émergeants: les processus de transfert, la recherche d'informations collaborative, les informations communiquées et les processus de recommandation.

**Conclusion:**

: Cette étude a montré que ce qu'on attendait n’était pas ce qui se passait réellement. Les exigences des politiques et des protocoles devaient être mises en œuvre efficacement pour améliorer les pratiques et les intégrer dans les directives biomédicales actuelles.

## Background

Maternal mortality is referred to as death caused by complications from pregnancy or childbirth. According to the current report by the WHO, there is a decline in the Maternal Mortality Rate (MMR) from 1990 to 2013 by 45% – from 380 deaths to 210 deaths per 100 000 live births. The decline does not necessarily meet the 5.5% rate required for the three quarters reduction targeted in the MDG goal 5 by 2015.^1^ Although all regions have shown significant advancement, the levels of maternal mortality in sub-Saharan Africa remain unacceptably high.^1^ In South Africa, the average MMR of 140 with a lower rate of 85 and 210 on the higher side indicates the need to implement further measures to reduce the lifetime risk of maternal death. Currently, the industrialized countries have a lifetime risk of maternal death of 1 in 4000, versus 1 in 51 in countries classified as ‘least developed’.^1^ Effective information communication during the management of labour and birth is an effective strategy to reduce maternal morbidity and mortality through identification of risks and abnormalities and promptly attending to them.^2^

Within the perinatal environment, interdisciplinary teams collaborate in providing quality care to women and the new-born during pregnancy, birth and the postpartum period with the ultimate goal of delivering a healthy mother and baby.^2^ The collaboration during perinatal care in addition to the management of obstetric emergencies allows the skilled birth attendants (SBAs) to intercept errors before they happen.^2^

The coordination of these collaborative activities requires information communication to be clear, timely, accurate, comprehensive and respectful in order to achieve cohesiveness within the perinatal setting.^3,4^ Fragmented activities, failure in systems and breakdown in communication contributes to a chain of actions that cause unexpected fatal outcomes.^5^

Improving maternal health requires health systems that function as an entity rather than silos with single interventions or actions by individual actors that form part of the activities of a team of actors.^6^ The complexity of management of perinatal care requires a variety of complex, yet comprehensive, interventions in order to reduce maternal mortality.^7^ The fact that complications can arise at any time and place requires single interventions which are comprehensive yet achievable. These interventions may be networked by several activities which require specific means of coordination. This, therefore, requires maternal health care which is dependent on appropriate health systems to have policies whose objective is dependent on each intervention to be achieved, whilst managing the complex interventions.^8^

Various factors were attributed to the expected information communication processes (ICP) within the obstetric units and between the referring facilities in the Cape Town Metro. The policies, protocols and guidelines are used as a means of coordination of activities in the management of the perinatal period. The definition of a protocol according to ^9^ is all steps in the management of a clinical condition which could cover the steps to develop a diagnosis or treat an illness. The protocols used in the study were used to describe the steps in diagnosis and management whilst describing the required processes to be followed in case of further management.

Guidelines are synonymously used with the term protocol emphasising guidance in the clinical management rather than ordering the course of action required. The practitioners are expected to use judgment in the care of patients. In an attempt to describe evidence-based health policy,^8^ it is important to know what is needed and to facilitate those needs, although that does not necessarily mean that the health is improved. Conducting studies on health policy process in especially low and middle-income settings, whilst focusing on the current activities, will allow for better identification of the content needed for the health policies.^8^

Regardless of whether the content of a guideline or a protocol reflects best practice, the usefulness and the impact is reflected by the adoption rate. Consequently, there is need for the developers of the policies, guidelines and protocols to contemplate the best representation or the guidelines for dissemination.^9^ Policies have to be contextualised in both context and place,^10^ meaning that the environment requiring policies is influenced by inter-organisational and network relationships. The purpose of the study was to understand the complexities involved during the ICP in the management of the intrapartum period. The review of what is expected in this study is based on the domestic policies, protocols and guidelines which guide the daily practices that determine what is actually happening within, and between, the health facilities.

## Methods

### Setting

The study was carried out in the Western Cape Province of South Africa. The province has two tertiary hospitals for obstetric referrals. Cape Town Metro has four intermediate level referral hospitals with 11 Midwifery Obstetric Units (MOUs) within the Metro District Health Services (DHS) managing low-risk women. The MOUs have the highest number of births, approximately 300 births per month, and are managed exclusively by midwives. The obstetric specialists are situated in the secondary and tertiary level hospitals managing intermediate and high-risk women. A multi-method qualitative case study approach with multiple units of analysis was utilised. Two case studies were purposefully selected from the Cape Metro from the MOUs with the highest number of deliveries per month. All the MOUs have specific referral criteria and management protocols for either intermediate or high-risk women when complications arise during labour and birth. The two selected MOUs in the study (referred to as MOU A and MOU B) were selected with the consecutive referring intermediate level hospitals (referred to as RH A and RH B).

### Data collection

Data were collected through conducting 24 interviews with the skilled birth attendants (SBAs) (doctors and midwives); eight midwives from MOU A, and six from MOU B; three doctors from RH A, one midwife, four doctors from referral hospital B and three midwives. These interviews explored activities influencing the ICP during the management of the intrapartum period. They were conducted in English lasting between 30 to 60 min as this was the medium of communication amongst other languages utilised, such as Afrikaans and Xhosa. All interviews were tape recorded with the permission of the participants and later transcribed and analysed thematically. The interviews were conducted in the health facilities at the convenience of the participants in a quiet room.

The document review of protocols, guidelines and policies was done in order to understand the expected ICP. These documents included: national maternal guidelines, provincial maternal guidelines, specific hospital protocols and the maternal case records (MCRs). The last-mentioned records include labour plans and partograms by identifying trends and patterns of the expected ICP. The partograms reviewed were those that were completed by the SBAs during the study observation period. During observation, the researcher assumed the role of the observer–as-participant, which involved observing and conducting short interviews regarding the observation. The researcher's role was overt, and the rapport created allowed the participants to freely speak about their experiences.

### Analysis

The researcher transcribed all interviews and analysed data using Atlas TI software 7. Furthermore, all documents were uploaded onto the Atlas TI and data coding was done. The thematic analysis was used to organise the emergent codes and categories in order to ensure rigorous analysis. The coded transcripts were read and reread whilst listening to the recorded tapes to identify emerging themes. Data were analysed according to each unit of analysis (MOU A, MOU B, RH A, and RH B). A report was created for the cases that considered the similarities and differences within, and between, the cases. Themes were created in consultation with the fellow researchers through discussion and amendment after the feedback.

## Ethical considerations

The research was approved by the review board and the ethics committee of the University Higher Degrees Committee (HDC) and the Western Cape Province, Department of Health, (RP052/2013) and the health facilities where the research was carried out. An informed consent was obtained from each interview participant. Ethical procedures were followed according to the ethics committee recommendations.

## Results

The main themes, expected ICP and the Actual ICP, were deduced from the objectives of the study, whilst the subthemes emerged inductively from the data coding and categorizing process.

### Theme expected information communication processes: Means of coordination

The policies, protocols and the maternal guidelines, which included SA national maternity guidelines and Western Cape guidelines, were reviewed. These included: the Western Cape level of care document, Metro West and Metro East protocols and referral criteria, neonatal protocols and referral criteria for the Cape Metro. The review revealed the expectations for the SBAs to ensure accurate and appropriate ICP whilst managing the women in the perinatal period. The subthemes that emerged from the data included accessibility, recommended tools for communication, the expected documentation, expected communication and referral criteria.

### Expected accessibility of obstetric services

The need for accessibility of obstetric services was described as being able to provide women with 24 hour care during pregnancy, birth and motherhood. Appropriate ICP ensures a swift reliable access to care especially during referrals:

Ensure 24-hour access to functioning emergency obstetric care (both basic and comprehensive).^1^

This accessibility to care was required from management at low-risk levels with the expectation that there are policies and protocols to ensure appropriate support between referring units.

### The expected documentation

The expected documentation included the data which was expected to be collected and the tools that were required to be used in the documentation. The early warning chart (EWC) was one of the tools required for use to recognise abnormalities and refer the woman:

Poor response to abnormal observations prompts an urgent need for the routine use of a national obstetric early warning chart used for all obstetric women …^11^

Situation, Background, Assessment and Recommendation (SBAR), which is a structured document, was also recommended to prompt an urgent need for referral and should also be used for consultation with referral hospital doctors, followed by appropriate training on the use of the tool:

… the SBAR communication format refers to the Situation, Background, Assessment and Recommendation format. It is important that the health care workers formulate an appropriate request and clearly document the response … Ensure training in the early warning charts and SBAR referral system is provided.^11^

The structured communication format is expected to increase the effectiveness of communication whilst formulating an appropriate request and documenting the response. Although most individuals used the SOAP (Subjective, Objective, Assessment, and Plan) criteria to hand over information, the recommended SBAR and the EWC have not been implemented and are in the process of implementation.

### Expected communication

Communication was expected to be effective, with tools such as radio, telephone and SBAR charts available between referring sites. Advice calls were recommended to be taken by doctors:

Effective communication system (radio or telephone) and reliable 24 hour transport service for emergency transfer to hospital … Ensure dedicated telephonic linkages for consultation for emergencies between referring and referral site are available. (SBAR charts).^12^

The expected communication requires intercollegial interactions to always be conducted in a respectful and professional manner. The communicator is required to be clear and upfront regarding the reason for calling the senior practitioner:

Intercollegial interactions should always be conducted in a respectful and professional manner.^13^

All data regarding the management of the intrapartum period was expected to be entered into the partogram with specific description of the required entry of information for progress of labour, condition of the mother and the condition of the foetus:

All observations pertinent to the progress of labour and the condition of the mother and the foetus during labour must be recorded on the partogram in the prescribed manner and intervals.^14^

Although the ISBAR was recommended for use during the referral of the woman, the partogram was also expected to be used for communication, and all information regarding the communication clearly written up on the reverse side of the partogram:

The professional taking action or phoning the next level of health management, must clearly document the actions on the reverse side of the observation chart …^12^

The expected communication required professionals to use precise, accurate and appropriate documentation in order to communicate effectively.

### Expected referral

The expected referral required a reliable mode of transport referral routes and appropriate communication processes. Protocols were required to streamline the routes whilst monitoring the referral processes:

A well-coordinated referral system, with access to transport and facilities, is essential for the provision of optimal care to all pregnant women in the district; it is essential to have in place a referral system with clear protocols of management, referral, transport and responsibility.^13^

An expected solution for mistakes during referrals was also suggested as the responsibility of the doctor at the referring level of care. It is her or his responsibility to contact the next level of care and communicate that information with the referring midwife. It was also recommended that the consultant in the higher level of care should resolve any disputes that arise during the referrals.

### Theme Actual information communication processes: Activities involved in the information communication processes

All SBAs viewed communication as essential for the coordination of work activities within each unit and had developed a culture that was understood by the other members. The handover process, collaborative information seeking, information communicated and referral processes were the categories emerging under this theme.

### Actual handover process in the midwifery obstetric units

The critical role of the handover process in the management of women during the intrapartum period was, by and large, the first statement expressed by midwives when asked about a day whilst on duty. The handover process involved the entry point for information exchange between shifts that enabled midwives to gain insight to the women already in the labour ward:

‘We go from bed to bed we discuss in detail if it's a post-partum woman, what has happened from when she came in through the labour, if there were any difficulties, focus on that, and when they leave you take over the woman and you go on.’ (P1:3)

All midwives and students are usually present during the handover process, as they are required to have information about each woman as they work through the day. The verbal handover is required to be documented in the maternal case record by the midwives handing over and those receiving the information:

‘… Ok in the mornings I take over with the rest of the staff so everybody, irrespective of whether you are working in the ante-natal clinic, we all take over in the labour ward. The reason why I do that is because I always feel that everybody should know what is going on you see because it might just happen that I need to call somebody for them to help me in the labour ward then they know what is going on in the labour ward.’ (P2:10)

The information exchange also referred to by midwives as ‘takeover’ also took place as the women arrived and progressed through the stages of labour. Being a midwifery-led unit that managed low-risk women, the midwives relied on phone calls for consultation, because there were no doctors in the MOU to consult when the midwives were unsure of how to proceed. The referral hospital was about 10 km away and, in case of a need for consultation, they would use telephones to call the hospital where the obstetric and paediatric specialists were located:

‘… No we don't have doctors in the MOU, we phone according to, say we have our whole criteria on where the woman needs to go, depending on the problem. Say if we find that the woman needs to go to RH A – which is mostly for the high-risk people. I consider level I as an intermediate that means the woman can be seen there and here.’ (P1:9)

Apart from the handover process, collaborative information seeking was noted during the management of the intrapartum period.

### Actual collaborative information seeking

The midwives cross-checked information that was given to be sure of the accuracy. Even though there was no specific allocation of a midwife per woman, the midwives worked as a team and kept each other informed of any new information regarding the women, including a particular woman's progress. This took place between the breaks for tea and lunches and also as they managed the women:

‘… Well I go and see and I check because there are times when the FHR is there and the progress is going slowly. The membranes are still intact and we need to see in 2 hours if this woman is dilated further or she stayed like that …’ (P8:23)

Using colleagues to record information when the midwife was busy allowed the midwife to write up information immediately, thus preventing loss of the required data. The use of non-medical personnel, such as the general assistants (GA), who were able to write down data was raised by the midwives. However, the question of whether or not the GAs wrote the correct information was not considered. GAs are unskilled staff who include cleaners or administrative assistants. The GAs would document the data on a piece of paper, which the midwife would later transfer to the maternal case record. The midwives also relied on memory to transcribe the notes:

‘But when we don't have students, we call out “weight” and someone will write it down but maybe she is busy while you are doing that and you ask her please to write it down on a piece of paper and will write 39, 47, blah blah blah.’ (P3:8)

In describing their day whilst working with women in labour, it emerged that the information collected by the midwives was part of the management of the intrapartum. The collection of information began with the assessment of the woman to determine when she was in labour:

‘Ok every admission that walks in here, we have got an admission bed and we do tests and urine, it's a routine we do for blood pressure, pulse, temperature and the h/b on admission. Then we do palpations and stretching of the abdomen, we palpate, we monitor the contractions, whether she is having contractions or not, as long as there no history of spontaneous rupture of membranes or if it's just a history of pains, even if you can't feel those pains we do the vaginal examinations so as to know where we stand.’ (P2:26)

The women were kept in the antenatal ward for four hours and, if diagnosed in labour, they would be admitted and managed in the antenatal ward during the latent phase of labour. During this time, the progress of labour was recorded in the maternal case record on the partogram. Those women who progressed to active phase > 4 cm dilated would be transferred to a labour bed and allowed to be mobile until delivery. All information collected during the management of labour was documented:

‘And when they are in active phase we prefer our women to walk around in the ante natal ward until they have the urge to bear down and when they reach the stage that they can't take the pain then we transfer them to the labour ward and then our trays are prepared in advance so that even if we don't have women that are in labour, whoever comes in with their head on the perineum, we have got a tray ready.’ (P3:24)

The midwives indicated that they worked as a team within the MOU. Even though the ward was busy, everyone was aware of what was happening in the ward as they would inform each other, or the midwife would examine the women to evaluate how far they were in labour. Sharing tasks was also common amongst the midwives in order to deal with the busy wards.

### Actual information communicated

The information communicated in the referral hospital is usually performed quickly, especially during emergency situations. The midwives from the MOU A would give the information to the doctors in order for them to decide whether to refer a woman to an intermediate risk or a high-risk hospital. The midwives would immediately explain the history of the woman as well as any concerns they had about her:

‘Sisters give me the whole information and what is the history of the woman?’ (P8:59).

‘Give us the meaty information so we can decide whether they need to come in or go there, can they stay there or do they need to go to RH B.’ (P10:10)

Disparities on which information was given, and prioritizing the information differed between the expectations of the doctors and what the midwives would give.

The specific information that is required to be communicated is important to prevent wasting of time and giving inappropriate information. The age, gravidity, the parity, and the reason for calling the referral hospital was deemed as important. The order in which the information was given differed from one midwife to another, and the doctors preferred the reason for calling as the most important information:

‘You need to say the background history of the woman, but they are very good at that – they will tell you the age, the gravidity/parity and how far the woman is, they are very good at that presentation but the important part is why you're phoning! [*Laughs*].’ (P10:36)

The age, gravidity, parity, investigations and the reason for calling, especially the current history, was information required to be communicated to the doctors by the midwives.

### Actual referral process

The referral process was emphasised as it was understood by both midwives and the doctors in the referral hospital. If a woman changes condition whilst in labour and becomes either a high risk or intermediate risk, they are expected to refer her in labour. Protocols for the Metro East are used to determine the types of women and where they are expected to be referred. When the midwives in MOU A phone the referral hospital they use the required criteria (in the protocol) to determine where they will send the woman. The protocols are adjusted according to the hospital capacity to accommodate the woman in labour:

‘… we phone according to, say we have our whole criteria on where the woman needs to go, depending on the problem … Then we discuss it with the doctor but we know already what kind of problem goes where …’ (P1:9)

When the MOU's call the referral hospital there are four MOU's that refer to RH A. The phone calls go through the switchboard and the doctors are contacted via intercom; they then contact the MOU. The doctors do not wait for the phone calls from the MOU as they have other tasks elsewhere in the hospital. The doctors prefer a hot line as the MOUs contact the hospital directly:

‘You can put it down to switchboard and say they can broadcast the call but they will put it through to their cell phone if it's after hours because after 10 pm there is no broadcasting of calls.’ (P11:32)

The consultation process requires the midwife in MOU A to give enough information for the doctor to make an informed decision. This happens when midwives encounter problems they are unsure of how to manage:

‘… I prefer them phoning if they need advice than sitting at that side not knowing what to do without a doctor that side, so I don't mind them doing that but not all doctors feel the same way.’ (P10:19)

The doctors’ work load in the referral hospital influences the ICP. There is no specific doctor allocated to answer the phone calls in the MOU. There are mainly two doctors on call who will answer calls and deal with the consultations and referrals as they come in. This means that the MOU A has to wait for the referral hospital to contact the doctors, whether in an emergency or merely for consultation purposes:

‘Yes there are two people … We are two of us on call and we will be taking calls and we will be seeing all the gynae women and all the obstetrics women, doing all the surgery as well. So there isn't a way for them to communicate with us other than the way that it is because as I said, it's not always us sitting in one place.’ (P10:64)

The fact that doctors can be anywhere in the hospital influences the flow of phone calls. The calls are directed to the area where the doctor is busy at that particular time. If the doctor is in theatre and scrubbed up the anaesthetist may receive calls and pass the information verbally to the doctor … However, the anaesthetist might not be conversant with obstetrics terminology, so that she or he will be unable to communicate the message from the midwives to the doctors properly:

‘If we are operating, scrubbed up or the anaesthetist needs to be our receptionist to convey the message, I think it's very frustrating for them as they mention it multiple times.’ (P10:5)

The doctors indicated that they mostly used the protocols to decide on women management and referral. Although there are protocols in place for the management of the intrapartum women, different doctors seemed to have different ways of managing the referrals and the consultations of the women.

## Discussion

### Means of coordination

The need to have policies, protocols and guidelines that can be easily adopted within a clinical setting is recommended.^8^ In the study, although most policies were made at the national level, the context within which they were applied required more specific approaches. The means of coordination should be specific to the interventions required, especially in the maternal health setting.^7^ As mentioned in the findings, the requirement for primary health care facilities to be accessible 24 hours allows low-risk women to have available services. The availability of the services is also linked to acceptability by the community and availability to the community.^10^ For accessibility to really have an impact on maternal heath, there is a need for a well-functioning system for accessing comprehensive emergency obstetric services.^10^ The accessibility of obstetric services emerged as the ‘umbrella’ of the other interventions such as expected documentation, recommended tools for documentation communication and the expected referral.

The recommended tools for communication had duplication of requirements where the information documented on the partogram was also required to be documented on the ISBAR and the EWC for every woman during the management of the intrapartum period. This tool required all information regarding the condition of the foetus, the progress of labour maternal condition and the management to be recorded. The effectiveness of use of the partogram evaluated in systematic reviews by^15^ reports low and middle-income settings recommendation for use of partograms.

Furthermore, the EWC was also recommended for use in detecting deviations from the normal during the management of the intrapartum period. This chart was also needed for communication. The tool SBAR was recommended for use during communication within and between referring centres. Although the structured communication document is recommended in literature for its effectiveness in communication,^16^ inappropriate use can lead to adverse outcomes. The duplication of information required in the three tools for documentation would increase the work of the SBAs in documenting information. Although telephones and radios were recommended for communication, the precise flow of information was not described in any of the guidelines. Specific guidelines are required in order to improve on all paper and electronic information tools to improve ICP

The expected communication was linked to the structure in the tools for communication. However, the need for specific protocols or communication guidelines is required to be drafted as context-specific, and training of the practitioners actualised during the implementation. The communication recommendations were mainly linked to referral requirements. The influence of documentation was not linked to communication processes but was seen as a separate entity.

### Understanding the actual information communication processes

The handover process within this study played the role of passing information regarding the management of perinatal women between SBA within and between shifts, which could be verbal and written summary notes.^17^ The communication was a culture that was considered important for transferring information between the team members, but the sufficiency of the information transferred was subjective to the receiver and sender. There was no structured communication between the SBAs. The use of nursing handover minimum data set and nursing notes for the specific context is recommended.^17^ There is a need to develop intrapartum midwifery-specific handover minimum data set and transcribed notes.^17^

The existence of finding, using and communicating the information in the study was complex-emphasised by the SBAs’ need to obtain information regarding the women in labour. The search for information was either between the experienced birth attendants, from protocols which they are familiar with, or other sources such as books and cell phones in the case of students. Information seekers, according to^18^ decide to seek information from a number of sources to choose from. The credibility, which refers to perceived trustworthiness, and utility described as usefulness, relevance, timeliness, accessibility and ease of use of the sources are characteristics described by^19^ as being relative to the perceptions and environments of the different users.

Although the SBAR tool was recommended for communication according to the means of communication, most of the SBAs were not aware of the existence of the SBAR, nor were they using the EWC. The partogram was heavily relied on for communication, and past history documented on the maternal case record. This is a very important tool for ICP, provided it is comprehensively completed and accompanies the woman in referral.

Protocols should be designed according to best evidence and, most importantly, should consider the context that includes the patient, treatment goals, local resources, staff and the local processes.^8^ The study highlighted the need for information communication protocols that are usable and understood by the intended users. Understanding work activities of the actors would enable development of appropriate means of coordination.

## Conclusion

What is expected by the means of coordination is not what is actually happening. The expected information communication is not the actual information communication. Context-specific culture for information collection, documentation and communication has allowed practitioners to use preferred processes in communicating amongst themselves. The ICP requires a structured process to be implemented with accurate monitoring and evaluation of the activities involved in the ICP. The author urges that the framework should provide a basis for the evaluation of the effectiveness involved in the ICP for the effective management of the intrapartum period.
